# Could Proteomics Become a Future Useful Tool to Shed Light on the Mechanisms of Rare Neurodegenerative Disorders?

**DOI:** 10.3390/ht7010002

**Published:** 2018-01-10

**Authors:** Maddalena Cagnone, Anna Bardoni, Paolo Iadarola, Simona Viglio

**Affiliations:** 1 Department of Molecular Medicine, Biochemistry Unit, University of Pavia, 27100 Pavia, Italy; maddalena.cagnone@gmail.com (M.C.); abardoni@unipv.it (A.B.); simona.viglio@unipv.it (S.V.); 2 Department of Biology and Biotechnologies “L. Spallanzani”, Biochemistry Unit, University of Pavia, 27100 Pavia, Italy

**Keywords:** rare neurodegenerative disorders, proteomics, biomarkers, 2-DE, LC-MS/MS

## Abstract

Very often the clinical features of rare neurodegenerative disorders overlap with those of other, more common clinical disturbances. As a consequence, not only the true incidence of these disorders is underestimated, but many patients also experience a significant delay before a definitive diagnosis. Under this scenario, it appears clear that any accurate tool producing information about the pathological mechanisms of these disorders would offer a novel context for their precise identification by strongly enhancing the interpretation of symptoms. With the advent of proteomics, detection and identification of proteins in different organs/tissues, aimed at understanding whether they represent an attractive tool for monitoring alterations in these districts, has become an area of increasing interest. The aim of this report is to provide an overview of the most recent applications of proteomics as a new strategy for identifying biomarkers with a clinical utility for the investigation of rare neurodegenerative disorders.

## 1. Introduction

As for all body systems, the nervous system (NS) is vulnerable to several afflictions that can result from genetic defects, physical damage (due to trauma or toxicity), infection, or, very often, simply from ageing. From a clinical point of view, all the disorders of the body nervous system are defined as neurological diseases and are characterized by a variety of symptoms. These span from altered levels of consciousness to problems of thought, feeling or behavior, cognitive deficits, confusion, poor coordination, loss of sensation, and paralysis [1,2,3]. Given the dramatically high number of people afflicted with slow progressive loss of one or more functions of the NS, the social impact of these illnesses is upsetting. The most common neurological disorders (i.e., Alzheimer’s or Parkinson’s disease) are accompanied by others, which affect only a small number of individuals and are regarded as being rare. Despite being serious or life-threatening disorders, patients that are affected are likely to go unrecognized if symptoms are mild or overlap with the more common clinical disturbances. The observation of these common clinical features in fact does not provide a rationale for discriminating a peculiar disorder from others, and, in the absence of unique symptoms, the unambiguous identification of these patients is often very difficult [4,5,6]. Thus, it cannot be expected to obtain the differential diagnosis of these disorders without performing a multidisciplinary clinical work-up, which may be expensive and time-consuming. Two important consequences result from this complicated picture. First, the true incidence of these disorders is underestimated. Second, many patients experience significant delay before a definitive diagnosis. Under this scenario, it appears clear that any accurate tool producing information about the pathological mechanisms of these disorders would offer a novel context for their precise identification by strongly enhancing the interpretation of symptoms [7]. The great deal of genetic research developed over the last few years has indeed provided progressive deepening of knowledge in this field. This improvement allowed for tricky details of the molecular mechanisms underlying the etiology and pathogenesis of several neurodegenerative disorders to merge. With the advent of proteomics, detection and identification of proteins in different organs/tissues, aimed at understanding whether they represent an attractive tool for monitoring alterations in these districts, has become an area of increasing interest. Substantial achievements have been obtained over the last decade in the screening of proteins as potential biomarkers of different diseases. Nevertheless, whether the application of proteomics (or its combination with genomics) would achieve decisive methodological progress in deciphering molecular mechanisms of these disorders remains a matter of speculation.

## 2. Outline of the Article

**Figure 1:**
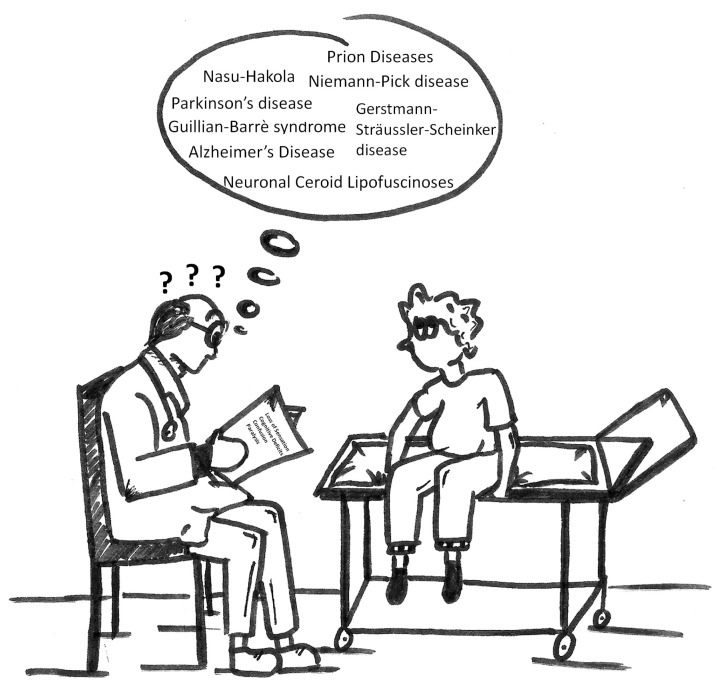
Cartoon showing the inability of physicians to a fast diagnosis of rare neurodegenerative diseases whose symptoms are similar to those of other more common neurological disorders.

The aim of this report is to provide an overview of the most recent applications of proteomics to the study of neurodegenerative disorders, which are catalogued as rare. As stated above, symptoms common to several neurodegenerative diseases may often lead physicians into confusion and cause considerable delay before a definitive diagnosis (Figure 1). The content of the following paragraphs supports the hypothesis that proteomics may provide an important contribution in deepening these pathologies.

Appropriate articles of our interest were selected through a search in the PubMed database [8]. The query placed was based on the terms “proteomics/complementary proteomics of rare neurodegenerative disorders; one-dimensional gel electrophoresis (1-DE); two-dimensional gel electrophoresis (2-DE); two-Dimensional differential gel electrophoresis (2D-DIGE); high performance liquid chromatography-mass spectrometry (HPLC-MS); combination of 2-DE with liquid chromatography-mass spectrometry (LC-MS) and capillary electrophoresis-mass spectrometry (CE-MS)” and it was restricted to full-text studies published during the last 10 years. All of the items found in the literature have been presented in this report.

## 3. Prion Disease

Prions are infectious agents that are responsible for a group of rare neurodegenerative diseases characterized by the aggregation and accumulation of misfolded proteins in brain tissue. Prions were initially identified in sheep as the causative agent of transmissible spongiform encephalopathies (TSE) derived from scabies, and, later, of bovine spongiform encephalopathy (BSE) [9,10,11]. Rare and fatal human prion diseases include sporadic/genetic Creutzfeldt–Jakob diseases (CJD); Gerstmann–Sträussler–Scheinker syndrome (GSSS); and, fatal familial insomnia (FFI). The etiology can be infectious, sporadic or hereditary. In any case the illness is progressive and always fatal, no effective medical treatment being available [12]. CJD associated with a specific mutation in the human prion protein gene is the most common prion disorder. Human-to-human transmission of prions, while being rare, has been related to a prion contamination during surgical procedures (tissue transplantation) or blood transfusions [13].

Several scientists have described changes in protein profiles of brain tissue from patients with prion disease [14]. For example, isobaric tags for relative and absolute quantitation (iTRAQ), combined with multidimensional LC-MS/MS, was used by Shi et al. [15] to investigate the global protein alterations in the cortex and cerebellum of sporadic and genetic forms of CJD, FFI, and controls. The iTRAQ-labeled products obtained after protein digestion and labeling were submitted to fractionation by both reverse phase-high performance liquid chromatography (RP-HPLC) and strong cation exchange (SCX) and peptide content was evaluated by matrix-assisted laser desorption ionization-time of flight (MALDI)-TOF/TOF. Among the 2287 proteins identified in the tissues analyzed, the total number of up- and down-regulated proteins was higher in cerebellum than in cortex regions (727 and 312 proteins, respectively) in both sporadic/genetic CJD and FFI patients. Gene Ontology (GO) analysis confirmed that the top five of the affected biological functions, including cellular component, biological process, and molecular function were completely identical in the tested samples of parietal lobe and cerebellum from sporadic CJD (sCJD), FFI, and G114V genetic CJD (gCJD) cases. The Kyoto Encyclopedia of Genes and Genomes (KEGG) analysis highlighted that pathways, such as oxidative phosphorylation, lysosome, and proteins, export had significantly changed. This said, it could be observed that, although the etiological agents and the pathogenesis may differ, the global proteomic profiles in brain tissues of the three human TSEs at terminal stage were very similar to each other. These findings allowed for understing that brain tissues from patients affected by these prion diseases had almost coincident biological functions.

iTRaQ-based proteomics (and KEGG pathway analysis) was also used by Wang et al. [16] to underline the importance of synaptic vesicle (SV) cycle pathway in providing an initial approach to study inborn errors of neurotransmitters. They compared the proteome of cerebrospinal fluid (CSF) from 20 patients affected by sCJD with that of 13 healthy subjects. Protein fragments were labeled using the 8plex iTRAQ reagent kit and analyzed with a nano-HPLC-MS system. Among the 1670 validated proteins, 557 were up-regulated and 595 were down-regulated in patients. Particularly interesting appeared 14 up-regulated proteins that participated in SV cycle pathway (i.e., AP2A1, SYT1, SNAP25, STXBP1, CLTB, Rab3a, and others). These are the key proteins that are modulating exocytosis and endocytosis within the SV cycle. The fact that they are also associated with other neurodegenerative diseases suggests that SV trafficking is a vulnerable target.

The global proteomic alterations in the cerebellum of the two most prevalent sCJD subtypes (MM1 and VV2) was explored by Tahir et al. [17], applying two-dimensional gel electrophoresis (2-DE), coupled with MS. 40 proteins were differentially expressed in MM1 and 43 in VV2. Twelve of these, common to the two groups, were associated with oxidative stress, thus suggesting the potential role of this process in the pathophysiology of sCJD. This platform of data convinced the authors to recommend the use of antioxidative therapeutic strategies to decrease the progression rate of sCJD.

Taken together, these studies demonstrate the importance of proteomics in this field. The discovery of a high number of changes in protein profiles of human prion diseases, particularly in sCJD, may in fact provide useful clues in the search for potential biomarkers to make an early diagnosis, assess disease progression, and monitor the effects of drugs during treatment trials.

## 4. Nasu-Hakola Disease

Nasu-Hakola disease (NHD), or Lipomembranous Osteodysplasia with Sclerosing Leukoencephalopathy (PLOSL), is a rare and fatal recessively inherited disorder. Its clinical course consists in four sequential stages involving the development of systemic bone cysts and progressive presenile dementia that is associated with sclerosing encephalopathy [18,19,20,21,22,23]. Given the common traits between Alzheimer’s (and other neurodegenerative disorders) and NHD, this rare pathology can be differentially diagnosed only by performing genetic analyses. These allowed to observe that the disorder results from the loss-of-function of two genes (*TYROBP* and *TREM2*), which encode for different subunits of the same membrane receptor signaling complex that plays a significant role in antigen-specific immune responses by B cells in vivo [20,22,24,25].

A novel context for the precise diagnosis of NHD would obviously come from the identification of protein biomarkers. However, to the best of our knowledge, the only proteomic profile available so far is that generated in our own laboratory on lymphoblastoid cells of 12 individuals [26]. Proteins from two patients with *TREM2* homozygous mutation, four patients with heterozygous mutation, and six healthy controls were separated by 2-DE and gels compared. 907 ± 41 spots were detected in controls; 886 ± 38 in heterozygotes and 897 ± 39 for homozygotes. 21 spots, which were common to the three groups, attracted our attention because of their significant differences in intensity and were identified by nLC-MS/MS. Most of them were enzymes involved in glucose metabolism (50%) or proteins implicated in regulation (40%). Only a small proportion (around 10%) was made of proteins involved in cytoskeletal and transport. The fact that a good number of these proteins intersected with those identified in other neurodegenerative disorders (i.e., Alzheimer’s, Parkinson’s diseases, and others) established the rationale for understanding the similarity in clinical characteristics of these patients. This study allowed for speculating that changes in glycolysis and gluconeogenesis enzymes could be responsible of major alterations in the energy metabolism pathway, which may explain puzzling symptoms of NHD patients. Further studies will clarify whether the alterations in glycolysis-related proteins are a cause or a consequence of the disease process.

## 5. Guillain-Barrè Syndrome

Guillain-Barré syndrome (GBS) is an autoimmune neuropathy targeting myelin sheaths and axons of the peripheral nervous system. It is the main cause of the acute neuromuscular paralysis and has an annual incidence ranging from 0.81 to 1.89 cases per 100,000 people [27,28]. The disorder is characterized by ascending limb weakness, mild sensory loss, and hyporeflexia or areflexia that progresses for up to four weeks before reaching a plateau. A proposed classification of GBS is based on clinical and neurophysiological findings: acute inflammatory demyelinating polyradiculone uropathy, acute motor axonal neuropathy, acute motor, sensory axonal neuropathy, and Fisher syndrome [29]. The mortality rates are 3–7% but permanent neurological sequelae affect about 7–15% of patients [28,30]. Recent studies have shown that, following infection by *Campylobacter jejuni* or cytomegalovirus, about 25% of patients suffer for the production of antibodies cross-reacting with gangliosides and other glycolipids leading to myelin destruction and consequent nerve conduction failure [4,28,29,30,31,32]. It has also been suggested that molecular mimicry between microbial antigens and host tissue could better clarify the pathogenesis of the disorder [33]. The first proteomic approach providing new insights into the pathological mechanisms underlying GBS was published by Lehmensiek et al. [34] who analyzed CSF from six patients and 12 controls by 2D-difference gel electrophoresis (2D-DIGE). Prior to the electrophoretic analysis, CSF samples were desalted and processed by affinity chromatography to extract the high abundant proteins IgG and albumin that could mask brain-specific proteins present at low-concentration in CSF. Analysis by MALDI-TOF MS of spots showing more than twofold difference between GBS and controls allowed for the identification in patients of 12 distinct proteins. While half of these (caldesmon 1 isoform, UDP glucose-hexose-1-phosphate uridyltransferase, heat shock protein 70, amyloidosis patient HL-heart-peptide, transferrin, and transthyretin isoforms) were down-regulated in GBS, the other half (serine/threonine kinase 10, alpha II spectrin, IgG heavy chain, SNC73 protein, cathepsin D preprotein, haptoglobin, and α-1-antitrypsin isoforms) were up-regulated. Several of the candidate proteins identified (i.e., haptoglobin, α-1-antitrypsin, transthyretin, and transferring) have been described in other inflammatory neurological diseases i.e., viral meningitis and multiple sclerosis and may be of limited value as biomarkers in GBS. The role of other identified candidates (i.e., serine/threonine kinase 10, GALT, alpha II spectrin, cathepsin D preprotein, caldesmon, HSP 70, amyloidosis patient HL-heart-peptide) still remains a matter of speculation and needs to be clarified.

The above technique, also applied by Jin et al. [35] to produce the protein pattern in CSF of GBS patients, resulted in the identification of six proteins whose levels were significantly altered in patients when compared to controls. Haptoglobin, apolipoprotein A-IV, and an unknown protein (PRO2044) were up-regulated, whereas transthyretin, apolipoprotein E, and fibrinogen were considerably down-regulated. All of the proteins are involved in inflammatory processes and were thus claimed to be new markers of the disorder even though their role needs to be evaluated by further studies.

2-DE and MALDI-TOF MS allowed for Chang et al. [36] to observe high levels of haptoglobin and apolipoprotein A-IV in the protein profile of CSF from GBS patients. Also, orosomucoid 1 was up-regulated in patients, whereas prostaglandin D2 synthase and transthyretin were down-regulated. Also, in this case, the role of protein identified needed a further evaluation.

The 2-DE proteomic profile of CSF from multiple sclerosis (MS) patients was compared by Fiorini et al. [37] to those of patients that were affected by other neurodegenerative diseases, including GBS. A significant up-regulation of 14.3.3 proteins was observed in MS. This is a family of seven isoforms (particularly abundant in the nervous system), which share a high degree of homology. In addition to their role as activators of neurotransmitter synthesis, these proteins are involved in many cellular processes, such as signaling, cell growth, division, adhesion, differentiation, and apoptosis. They have been indicated as possible markers of axonal damage or inflammation. As suggested by other authors, also the down-regulated proteins can be considered putative biomarkers for GBS.

In this context, the work by Yang et al. [38] is of great interest. By analyzing (with 2-DE and MALDI-TOF/TOF MS), the CSF proteome of eight GBS patients, and 10 controls affected by other neurological disorders they observed that six spots had significant differential expression between the two groups. The most significant changes concerned the down-regulation of cystatin C (a member of the superfamily of cysteine-protease inhibitors), which is involved in the pathogenesis of cerebral amyloidosis. This behavior in GBS patients had never been reported before and could be suggestive of a possible role of cystatin C in the pathological mechanisms of the disease.

Aim of Sawai et al. [39] was identification of the possible immunologic target molecule of pathogenic autoantibodies in patients with cytomegalovirus (CMV)-related GBS demyelinating form. The proteins extracted from schwannoma cell line YST-1 were separated by 2-DE, transferred to polyvinylidene difluoride (PVDF) membrane and immunoblotted with patient’s sera. Six proteins were identified under the eight immunoreactive spots. Among the other candidates, only membrane-organizing extension spike protein (moesin) was a plasma membrane protein expressed by Schwann cells thought to be crucial for myelination processes. By using the BLAST program [40], the molecular mimicry between moesin and the CMV protein “phosphoprotein 85” was also established, thus confirming its role as a possible immunologic target molecule of pathogenic autoantibodies.

Light on the molecular mimicry between *C. jejuni* and proteins from human peripheral nerve (HPN) tissue was shed by the work of Loshaj-Shala [41]. Proteins that were extracted from *C. jejuni* and HPN were separated by sodium dodecyl sulfate polyacrylamide gel electrophoresis (SDS-PAGE) and their immunogenic reactivity towards sera from GBS patients determined by western blotting. The electrophoretic bands showing potential cross reactivity were isolated, proteins extracted, digested, and submitted to nano-high performance liquid chromatography-electrospray ionization-high resolution mass spectrometry (nHPLC-nESI-HRMS). The results showed that *C. Jejuni* chaperone protein DnaK and human peripheral nerve HSP 70; bacterial GroEL and human HSP 60 share high sequence homology. These findings strongly suggest that HSP chaperones could be potentially involved in the development of the autoimmune response associated to GBS.

Piccolo is a high molecular weight protein that is involved in assembling presynaptic F-actin, gathering synaptic vesicles, and controlling synaptic transmission and voltage-gated calcium channel function. Being also involved in multiple protein-protein interactions and functional associations, Piccolo has a fundamental role in multiple biological processes, such as regulation of exocytosis, synapse assembly, regulation of neurotransmission, cytoskeleton organization, and sensory perception [42]. Since the presynaptic neuromuscular junction (NMJ) has been considered a potential target to autoimmune attack in GBS, Piccolo may be involved in remodeling mechanisms of the presynaptic active zone during recovery from the acute phase of the disease. By analyzing serum from GBS patients (with 1-DE coupled with MS/MS), Mateos-Hernández et al. [43] showed an increase of Piccolo concentration, thus suggesting that Piccolo protein could be considered a potential serological marker of disease recovery. The authors used STRING [44] for the in silico characterization of Piccolo-protein interactions using a high confidence interaction score (0.700). Protein ontology analysis was performed using the Blast2GO software [45].

Marching to a different drummer compared to other authors, recently Ziganshin et al. [46] subverted the above-mentioned infection-triggered hypothesis for the pathogenesis of GBS demyelinating form. They suggested that an autoimmune response is not required for the peripheral nerve conductivity damaging to start. Their proteomic work was focused on CSF peptides isolated after solid phase extraction from GBS, multiple sclerosis (MS), patients with meningitis, and controls affected by non-neurological diseases. The authors showed that CSF was enriched with peptides related to the proteins that are involved in the arrangement of the axonal domains in GBS patients only. The up-regulation of CSF cytokines was associated with innate immunity linked to the defensive responses to bacteria. According to these findings, the authors concluded that the autoantibody production could be an optional secondary process following primary peripheral nervous system damaging initiated as an innate immunity-associated local inflammation. An accurate analysis of the protein profiling of CSF from GBS patients was performed by D’Aguanno et al. [47] applying 2-DE, followed by MALDI-TOF/TOF. A few inflammation-related proteins (vitamin D-binding protein, β-2 glycoprotein I (ApoH), and a complement component C3 isoform) were up-regulated in patients when compared to controls, whereas transthyretin, apolipoprotein E, albumin, and five of its fragments were down-regulated. Being these proteins already described in other neurological diseases, the authors concluded that that they cannot be used as biomarkers in GBS. An isoelectric focusing dinitrophenylhydrazine-based technique was also used to analyze the extent of protein carbonylation resulting from an oxidative damage. The major sensitivity to carbonylation observed for albumin and α-glycoprotein in inflammation, suggested that oxidative stress could contribute to the immunopathological mechanisms in GBS.

## 6. Niemann-Pick Disease

The eponym Niemann-Pick disease (NPD) refers to a group of rare, autosomal recessive disorders that are characterized by lipid storage and foam cell infiltration in tissues. The typical clinical features of patients affected include hepatosplenomegaly, pulmonary insufficiency, and/or central nervous system (CNS) involvement [5,6,48]. Two distinct metabolic abnormalities account for NPD. The first is the deficiency of the enzyme acid sphingomyelinase (ASM) that plays a key role in maintaining sphingolipid homeostasis and participating in membrane turnover. Patients with ASM deficiency are classified into type A and B NPD. Type A patients are characterized by hepatosplenomegaly in infancy and profound CNS involvement. They cannot survive beyond two years of age. Conversely, type B patients, while showing hepatosplenomegaly and lung pathologic alterations, do not have CNS involvement [48].

The second results from mutations in genes encoding for two distinct cholesterol-binding proteins (NPC1 and NPC2). Patients showing this metabolic alteration are classified into type C NPD (NPC) and have mild hepatosplenomegaly, together with a deep involvement in their CNS showing symptoms ranging from ataxia and dystonia to dementia. Mutations in the NPC1 protein are implicated in 95% of patients with type C NPD disease.

To achieve a deeper understanding of the early NPC pathological processes and to identify a set of differentially expressed proteins that may be used as biomarkers, Cologna et al. [49] used 2-DE followed by both MALDI-TOF/TOF and LC-ESI-MS/MS to analyze CSF from 42 NPC1 pediatric patients and 30 age-matched controls who were undergoing CSF collection for another clinical indication. When compared to controls, altered expression of glutathione S-transferase α, superoxide dismutase, and fatty acid binding protein (FABP) was found in CSF of NPC1 patients. Interestingly, CSF of a subset of NPC1 patients on miglustat (a glycosphingolipid synthesis inhibitor), showed significantly decreased levels of FABP3 as compared to patients who were not undergoing miglustat therapy. Also, in this case, proteins identified warrant further analysis to better understand the exact mechanisms by which they affect the NPC1 phenotype.

The most prevalent mutation in NPC1 protein is the missense mutation I1061T that is occurring in about 15–20% of the alleles [50,51]. An isobaric labeling-based quantitative proteomic approach to identify proteins with relevance to NPC pathogenesis due to this mutation was applied by Rauniyar et al. [52]. Fibroblasts from healthy (NPC1WT) and patients (NPC1I1061T) were labeled with different isotopic variant of an amine-reactive six-plex tandem mass tags (TMT) isobaric reagent. They were combined and analyzed by multidimensional protein identification technology (MudPIT). An overview of the biological significance of the differentially expressed proteins was done performing a Gene Ontology (GO) enrichment analysis with the Ontologizer software tool [53]. The identification of 281 proteins that are associated with different metabolisms (ROS process, antioxidant activity, steroid metabolic process, lipid localization, apoptosis, energy metabolism) that were significantly altered in NPC1I1061T fibroblasts when compared to NPC1WT provided a starting point for future investigations.

Only a limited number of NPC1 binding proteins (Npc1-BPs) has been identified so far. This number was increased by Macias-Vidal et al. [54] who applied LC-MS/MS to identify 31 new lysosomal NPC1-BPs associated to: (i) proteolytic and homeostatic processes; (ii) lipid catabolism; (iii) vacuole organization and (iv) cation transport. The authors focused their attention on cathepsin D, which is an endopeptidase involved in protein degradation, apoptosis and autophagy, intracellular sphingolipid metabolism, and cholesterol trafficking. The observation that cathepsin D activity was reduced in NPC fibroblasts from NPD patients led the authors to consider its alterations closely correlated with the pathogenesis of the disease. This opened the door to the understanding of the mechanisms involved in the triggering and progression of NPD.

Engineered super paramagnetic iron oxide nanoparticles (SPIONs) targeting distinct subcellular compartments have been developed by Tharkeshwar et al. [55]. They internalized and accumulated dimercaptosuccinic acid-coated SPIONs in late endosomes/lysosomes, and inserted aminolipid-SPIONs in the plasma membrane. The aim of this work was standardization of magnetic isolation procedures for these membrane compartments. The approach was validated by comparing the biomolecular composition of lysosomes and plasma membranes isolated from wild-type and Niemann-Pick disease type C1 (NPC1) deficient cells. The separation of lysosome and plasma proteins by SDS-PAGE, followed by their analysis with LC-MS/MS showed a significant up-regulation in NPC1 proteins linked to autophagy and lysosomal catabolism reflecting vesicular transport obstruction and defective lysosomal turnover in patients affected by NPC1.

The pharmacological activity of methyl-β-cyclodextrin (MβCD), a drug that reduces lysosomal cholesterol accumulation in fibroblasts from NPC1 patients was analyzed by Li et al. [56] using 1-DE followed by LC-MS/MS. Briefly, wild-type and NPC1 patients’ skin fibroblasts were incubated with three preparations of MβCD, from two commercial vendors, characterized by different average molecular weights and levels of methylation in side chains. The protein list for all analyzed pathways and processes was obtained from the KEGG pathway database. Their functional annotations were manually confirmed using the UniProtKB protein database, the NCBI protein database or available information from the literature. The proteomic profiles showed that higher a level of methylation is likely to be more efficient in ameliorating dysregulated protein expression in NPC1 disease cells, including proteins involving lysosome formation, vesicle expansion, and completion during autophagy, SNARE proteins interaction in vesicular transport, and steroid biosynthesis. This new proteomic approach has an important clinical relevance as it may be used as a quality control method for selection of cyclodextrins to be used as therapeutic agents for treatment of NPC and/or other lysosomal storage diseases.

Subramanian et al. [57] used amine-reactive TMT to perform a differential labeling and to quantify the proteome in NPC1 I1061T cells treated in the absence or presence of Vorinostat, a histone deacetylase inhibitor restoring cholesterol homeostasis in fibroblasts from NPC patients. The labeled samples, as analyzed by MudPIT [58], allowed for observing that the treatment of NPC1 I1061T fibroblasts with Vorinostat enhanced pathways promoting the folding, stabilization and trafficking of NPC1 mutant protein to lysosomes. The biological function of the differentially expressed proteins was analyzed by mapping against the KEGG pathways. Their results confirmed that Vorinostat modulated the expression of lysosomal acid lipase, which is a protein mediating cholesterol efflux in NPC1 I1061Tfibroblasts. Based on these data, the authors concluded that Vorinostat may provide a useful framework for a broader range of therapeutic intervention strategies for clinical treatment of NPC.

Surprisingly, proteomic studies concerning type A and type B NPD have not been reported yet.

## 7. Neuronal Ceroid Lipofuscinoses

Neuronal ceroid lipofuscinoses (NCLs) are a group of severe autosomal recessive neurodegenerative disorders affecting children and young adults, collectively known as Batten disease [59,60,61]. From a clinical point of view, these disorders are characterized by common features including early onset of vision loss and sleep disorders, seizures, loss of motor function, leading to eventual quadriplegia, progressive development of mental retardation, behavioral changes, and early death.

The main pathological hallmark of NCL is the massive loss of brain size at autopsy due to neuronal loss and the storage in neuronal tissues of a typical autofluorescent pigment called ceroid lipofuscin. The earliest literature defining the clinical and pathological features of NCL was generated on the basis of the age of clinical onset (Infantile, Late-Infantile, Juvenile, and adult NCL), or of the nature of the storage material (GROD body, curvilinear body, or fingerprint body storage diseases). The molecular analysis allowed for discovering at least 10 distinct genetic loci that result in different types of NCL with clinical and pathological overlapping.

To investigate the molecular basis of lysosomal storage diseases (LSD) with unsolved or ambiguous etiology, Sleat et al. [62] carried out a comparative proteomic analysis of purified mannose 6-phosphate glycoproteins. These were obtained from brain autopsy of six normal controls and 27 LSD patients, 17 of which were affected by different forms of NCL and analyzed by 1-DE coupled with LC-MS/MS. New atypical variants of unrelated lysosomal diseases that were diagnosed as adult NCL (ANCL) were identified. In a few ANCL patients, the lack of CLN5 protein, normally absent in the late infantile form of the disease, was also observed. The authors concluded that the proteomic methods for protein expression profiling represent a promising approach for the investigation of lysosomal storage diseases of unknown or ambiguous etiology.

Wang et al. [63] identified and characterized differentially expressed proteins in fibroblasts derived from skin biopsies of controls and NCL patients with mutations in *CLN1*, *CLN2*, *CLN3*, or *CLN8* genes by applying a two-dimensional protein fragmentation (PF2D) study. Analysis of functional enriched categories was carried out on the differentially expressed proteins, using the GO database with DAVID program [64]. Among the 40 differentially expressed spots that were selected on the PF2D maps, the authors identified (by MALDI-TOF-MS or LC–ESI-MS/MS) 24 proteins that can be clustered into categories of intermediate filaments, cell motility, apoptosis, cytoskeleton, membrane trafficking, calcium binding, nucleosome assembly, pigment granule and cell development based on GO categories. These findings suggested a possible cell cytoskeleton and cell motility breakdown in patients with NCL1, 2, 3, and 8 during the NCL development. Furthermore, the differentially expressed proteins may represent NCL-specific biomarkers for the disorder.

2-DE followed by MALDI-TOF/TOF was also used by Haddad et al. [65] to highlight the interaction between CLN5/CLN8 proteins and their role in sphingolipid metabolism. Their findings suggest that, most likely, both of the proteins are positive modulators of (dihydro)ceramide synthase 1 (CerS1) and/or CerS2. Furthermore, defects of CLN5/CLN8 affect the metabolism of C18:0/C24/C24:1 ceramide species and defects of CLN5 are corrected by CLN8. These results were a strong body of evidence that these proteins could be functionally related.

Scifo et al. [66] mapped the protein interaction network in human neuroblastoma cells to better understand the mechanistic and biological functions of human ceroid-lipofuscinosis neuronal protein 3 (CLN3) in the brain of patients affected by juvenile NCL (JNCL) or CLN3 disease. Tandem Affinity Purification-Mass Spectrometry (TAP-MS) combined with Significance Analysis of Interactome (SAINT), allowed for the identification of 58 CLN3 interacting partners (IP), 18 of which were also CLN5 IP. The combined CLN3-CLN5 interactome suggested the involvement of CLN3 in transmembrane transport, lipid homeostasis, neuronal excitability, G-protein signaling, and protein folding/sorting in the endoplasmic reticulum.

The same research group analyzed the palmitoyl protein thioesterase 1 (PPT1 or CLN1 protein) interactome in SH-SY5Y human neuroblastoma cells [67,68]. PPT1 is a hydrolytic enzyme, encoded by CLN1 gene, cleaving off long fatty acid chains, i.e., palmitate, from modified cysteine residues of proteins. Depalmitoylation is responsible for the release of synaptic proteins from the membrane, so that they can participate in vesicular trafficking. Patients that are affected by mutations in the CLN1 gene develop a rapidly progressive neurodegenerative disorder, also known as CLN1 disease. The authors used a single step affinity purification approach coupled to MS (AP-MS) to probe human PPT1 IP in neuronal cells of the brain. Briefly, protein complexes were isolated from human PPT1 expressing SH-SY5Y cells, submitted to filter-aided sample preparation (FASP), and analyzed by MS. A total of 23 PPT1 IP were identified from label free quantitation of the MS data by SAINT platform, including neurodegenerative disease causative proteins and mitochondrial proteins (pyruvate dehydrogenase and mitochondrial ATP synthase complexes). Three of the identified PPT1 IP, collapsin response mediator protein 1 (CRMP1), dopamine β-hydroxilase (DBH), and microtubule-associated protein 1B (MAP1B), are thought to be palmitoylated, thus suggesting a key role of PPT1 in the regulation of their intercellular localization by depalmitoylation at various stages during neuronal migration, axon guidance, and dopamine receptor mediated signaling.

2D-DIGE followed by MS was used by Hersrud et al. [69] to identify candidate biomarkers in blood plasma of NCL patients. 17 healthy individuals, 13 patients affected by JNCL, three diagnosed with infantile NCL (INCL), one diagnosed with late infantile NCL (LINCL), and two affected by the Finnish variant late infantile NCL were analyzed. Seven differentially expressed candidate biomarkers (BDNF, NrCAM, clusterin, adiponectin, apoE, VCAM-1 and myoglobin) associated with neurodegeneration and/or brain injury and reflecting a systemic disease state could be identified. The authors concluded that this study may be of great clinical usefulness for assessing effectiveness of therapy by measuring the baseline levels of these identified biomarkers and progressively tracking their levels.

Recently, mutations in the *DNAJC5*/*CLN4* gene, encoding the presynaptic co-chaperone CSP, have been claimed to cause autosomal-dominant NCL. The quantitative proteomic approach (based on LC-MS/MS) applied by Henderson et al. [70] aimed to determine the consequences of the accumulation of PPT1/CLN1, which is a CLN4-associated protein responsible for the depalmitolylation of CSP. Protein interactions and functions of significant proteomic hits were analyzed using the QIAGEN Ingenuity Pathway Analysis software (Redwood, CA, USA). The comparison, by quantitative proteomics, of the palmitomes from control and DNAJC5/CLN4 patient brains uncovered global changes in protein palmitoylation, mainly involving lysosomal and synaptic proteins. These findings provided new insights into the pathogenesis of NCLs and suggested that aberrant protein palmitoylation may be critical in the etiology of neurodegeneration.

The list of diseases considered in this report together with the methodological approaches that are applied for their study, the type of samples considered, the number of proteins identified and the original article of reference, is reported in Table 1.

## 8. Pros and cons of Techniques Mentioned in this Report

As shown in the above paragraphs, investigation of disorders considered in this report was done using a good number of gel-based and/or gel-free approaches. The application of such a variety of techniques can create confusion to the reader who could ask which is the rationale for choosing a method. These articles demonstrate that no fixed protocol applicable to all of the samples does exist. The procedure selected depends primarily on the nature and complexity of the specimen to be characterized, on the specific classes of proteins to be extracted and on the focus of the proteome analysis. Regardless of the choice of the method, advantages and disadvantages are associated to each given proteomic separation technique. Pros and cons of the techniques that are encountered in this paper have been summarized in Table 2.

## 9. Conclusions

The constantly growing production of excellent proteomic articles dealing with rare neurological disorders confirms that this methodological tool may become decisive for the identification/characterization of disease-associated proteins. The discovery of biomarkers of rare neurological disorders is still in early development and findings mentioned above still need extensive validation. However, the development of novel platforms to demonstrate their clinical utility will probably indicate proteomics as a new strategy for investigating these disorders. With the rapid pace of technological improvements, this approach is attracting increasing attention and a significant impact in this area is expected in coming years.

## Figures and Tables

**Table 1:** List of diseases considered in this report together with the methodological approaches applied for their study, the type of samples considered, the number of proteins identified and the original article of reference.

Type of Neurodegenerative Disease	Type of Samples Analyzed	Number of Proteins Identified	Proteomic Approach	Ref.
Prion disease	Cortex and cerebellum	2287	iTRAQ, LC-MS/MS	[15]
	CSF	1670	iTRAQ, LC-MS/MS	[16]
	Cerebellum	83	2-DE, MS	[17]
Nasu-Hakola disease	Lymphoblastoid cells	21	2-DE, nLC-MS/MS	[26]
Guillain-Barré syndrome	CSF	12	2D-DIGE, MALDI-TOF MS	[34]
	CSF	47	2D-DIGE, MALDI-TOF MS	[35]
	CSF	6	2-DE, MALDI-TOF MS	[36]
	CSF	3	2-DE	[37]
	CSF	10	2-DE, MALDI-TOF MS	[38]
	Schwannoma cell line YST-1	6	2-DE	[39]
	HPN tissue	3	1-DE, nHPLC-nESI-HRMS	[41]
	Serum	330	iTRAQ, LC-MS/MS	[43]
	CSF	854	1-DE, LC-MS/MS	[46]
	CSF	17	2-DE, MALDI-TOF/TOF	[47]
Niemann-Pick disease	CSF	109	2-DE, MALDI-TOF/TOF, LC-ESI-MS/MS	[49]
	NPC1I1061T fibroblasts	4308	TMT labelling, MudPIT	[52]
	NPC fibroblasts	114	1-DE, LC-MS/MS	[54]
	NPC1 deficient cells	7342	1-DE, LC-MS/MS	[55]
	Human fibroblasts	19	1-DE, LC-MS/MS	[56]
	NPC1 I1061T fibroblasts	2916	TMT, MudPIT	[57]
Neuronal ceroid lipofuscinoses	Human brains	320	1-DE, LC-MS/MS	[62]
	Human fibroblasts	24	PF2D, MALDI-TOF-MS, LC-ESI-MS/MS	[63]
	Human fibroblasts	8	2-DE, MALDI-TOF/TOF	[65]
	Human neuroblastoma cells	58	TAP-MS	[66]
	Human neuroblastoma cells	23	TAP-MS	[67,68]
	Plasma	27	2D-DIGE, LC-MS/MS	[69]
	Human brains	17	LC-MS/MS	[70]

1-DE. one-dimensional gel electrophoresis; 2D-DIGE: two-dimensional differential gel electrophoresis; 2-DE: two-dimensional gel electrophoresis; HPLC: high-performance liquid chromatography; TRAQ: isobaric tags for relative and absolute quantitation; LC-ESI-MS/MS: liquid chromatography-electrospray ionization tandem mass spectrometry; LC-MS/MS: liquid chromatography-tandem mass spectrometry; LC-MS: liquid chromatography–mass spectrometry; MALDI-TOF: matrix assisted laser desorption ionization-time of flight; MS: mass spectrometry; MudPIT: multidimensional protein identification technology; PF2D: two-dimensional protein fragmentation; TAP-MS: tandem affinity purification mass spectrometry; TMT: tandem mass tag.

**Table 2:** Pros and cons of the techniques encountered in this report.

Technique	Pros	Cons
**1-DE**	- Allows separation of all types of proteins, even those insoluble in water.	- Overlapping of closely-spaced bands with consequent limited resolution. - Not able to identify isoforms and Low Molecular Weight proteins. - For protein identification needs the coupling of another technique, i.e., immunoblotting or MS.
**2-DE**	- Good resolution of protein mixtures.-Allows discernment of post-translational modifications. - Comparison of multiple gels facilitated by image analysis software. - More statistically robust than other methods - Use of broad/narrow pH gradients - Identification of protein isoforms - Cost-effectiveness of the procedure	- Unable to resolve low (<10 kDa) and high (MW > 250 KDa) molecular weight proteins - Unable to resolve highly acidic (pI < 3) or basic proteins (pI > 9) - The presence of high abundant proteins (i.e., immunoglobulins and/or albumin) masks the low abundant ones. - Final identification requires spot removal from gels, digestion and peptide analysis by MS. - Low throughput.
**2D-DIGE**	- Very sensitive. - Ratio of protein expression can be obtained in a single gel. - An internal standard can be introduced in each gel to reduce gel-to-gel variation.	- Also this technique shows all drawbacks previously underlined for 2 DE (see above).
**LC-MS**	- Fast and robust tool - Identification of membrane proteins - Identification of low abundant proteins - Powerful tool for studying large-scale protein expression and characterization of complex biological systems - Detection of proteins with extreme/peculiar molecular mass and pI - High sensitivity (sub-pM range) - High throughput	- Expensive, in terms of capital and running costs
